# The Relationship between Low Skeletal Muscle Mass and Subsequent Oral Intake Ability among the Aged Population

**DOI:** 10.3390/healthcare11050729

**Published:** 2023-03-02

**Authors:** Mari Nakao-Kato, Shin-Ichi Izumi, Shinta Nishioka, Ryo Momosaki, Hidetaka Wakabayashi

**Affiliations:** 1 Department of Physical Medicine and Rehabilitation, Graduate School of Biomedical Engineering, Tohoku University, Sendai 980-8575, Japan; 2 Department of Clinical Nutrition and Food Service, Nagasaki Rehabilitation Hospital, Nagasaki 850-0854, Japan; 3 Department of Rehabilitation Medicine, Mie University Graduate School of Medicine, Tsu 514-8507, Japan; 4 Department of Rehabilitation Medicine, Tokyo Woman’s Medicine University Hospital, Tokyo 162-8666, Japan

**Keywords:** skeletal muscle mass, functional oral intake, deglutition disorders, aged population, sarcopenia

## Abstract

This study aimed to determine the relationship between skeletal muscle mass in an aged population with limited oral intake upon admission and functional oral intake at the subsequent 3-month follow-up. Methods: This was a retrospective cohort study using the Japanese Sarcopenia Dysphagia Database involving older adults (≥60 years) with limited oral intake (Food Intake Level Scale [FILS] level of ≤8). People without skeletal muscle mass index (SMI) data, unknown methods of SMI evaluation, and SMI evaluation by DXA were excluded. Data for 76 people (47 women, 29 men) were analyzed (mean [standard deviation] age: 80.8 [9.0] years; median SMI: women, 4.80 kg/m^2^; men, 6.50 kg/m^2^). There were no significant differences in age, FILS upon admission and methods of nutrition intake between the low (*n* = 46) and the high skeletal muscle mass groups (*n* = 30), although the proportion of sex between the two groups was different. The FILS level at the time of follow-up differed significantly between the groups (*p* < 0.01). The SMI upon admission (odds ratio: 2.99, 95% confidence interval: 1.09–8.16) were significantly associated with the FILS level at the time of follow-up after adjustment for sex, age, and history of stroke and/or dementia (*p* < 0.05, power = 0.756). Conclusion: A low skeletal muscle mass is a disadvantage for achieving a subsequent fully functional oral intake ability among the aged population with limited oral intake upon admission.

## 1. Introduction

Nutritional disorders can occur because of dysphagia [1,2]. Namasivayam-McDonald and colleagues reported that, among residents of long-term care facilities, the odds of being malnourished were double in those who showed signs of dysphagia compared with those who did not have signs of dysphagia [3]. Shimizu and colleagues reported that patients who were admitted to the rehabilitation ward and served texture-modified food because of limited oral intake ability were likely to have a low skeletal muscle mass [4].

Whether the loss of skeletal muscle mass causes dysphagia and how and to what extent this might occur are unknown. Additionally, whether there is heterogeneity by sex in dysphagia caused by a loss of skeletal muscle mass is unknown. Furthermore, the proportion of skeletal muscle mass that contributes to the severity of dysphagia remains unknown. Four Japanese academic societies published a position paper in 2019 stating that dysphagia can occur because of sarcopenia itself without a specific disease that directly causes dysphagia. This condition was named sarcopenic dysphagia [5]. Pathologically, sarcopenia refers to the loss of muscle mass associated with aging [6]. Generally, sarcopenia is characterized by a decrease in the number of muscle fibers, and by a replacement with fat and connective tissue [7,8]. The European Working Group on Sarcopenia in Older People updated the definition of sarcopenia, and a diagnosis is made on the basis of the presence of low muscle quantity or quality [9]. An increasing number of studies have reported the age-related change in muscles related to swallowing. Yamaguchi and colleagues reported adipose tissue deposition in the tongue and suprahyoid muscle as evaluated by ultrasonography according to age, but this was not related to oral function [10]. They also reported a negative association of the age with muscle mass cross-section area in the same population [10]. Nakao and colleagues reported that fat infiltration of the tongue increased with aging, and it was correlated with tongue pressure, though it had no effect on swallowing physiology (aspiration, penetration, and pharyngeal residue) [11]. Sakai et al. [12] performed systematic review and meta-analysis to investigate the association of oral function and dysphagia in community-dwelling older adults with sarcopenia. They reported that older adults with sarcopenia had 5.40 kPa [4.17–6.62 kPa] lower tongue pressure in comparison to older adults without sarcopenia. However, their result was inconclusive on the relationship between sarcopenia and dysphagia. A study by Chen and colleagues [13] showed a change of hyoid velocity measured with ultrasonography and delay of 100 mL water swallowing time for sarcopenic individuals in the community aged 65 and over.

It should be noted that the physiological relationship between reduced skeletal muscle mass and dysphagia has not yet been fully clarified. Sugiya and colleagues reported the relationship between decreased tongue strength and the skeletal muscle mass index (SMI) in patients with chronic obstructive pulmonary disease, but there was no difference in the prevalence of dysphagia compared with the control group [14]. Nagashima and colleagues examined the relationships between maximum tongue pressure, the SMI, and posterior pharyngeal wall movement with outpatients aged ≥65 years old who complained about their dysphagia [15]. They found a negative correlation between the SMI and maximum tongue pressure, but posterior pharyngeal wall movement was not correlated with the SMI [15].

This study aimed to evaluate the relationship between skeletal muscle mass measured with the BIA method upon admission and the subsequent oral intake ability at the follow-up among older people with limited oral intake upon admission. We hypothesize that older people with limited oral intake and a low skeletal muscle mass upon admission or registration achieve a lower goal of oral intake at the time of follow-up (3 months after registration) than that of older people with high skeletal muscle mass upon admission.

## 2. Materials and Methods

### 2.1. Study Design and Database Used for Analysis

This was a retrospective cohort study using the Japanese Sarcopenia Dysphagia Database (JSDD). The details of this database were reported in a previous study [16]. The JSDD database was constructed by the Rehabilitation Nutrition Database Committee of the Japanese Association of Rehabilitation Nutrition and the Japanese Working Group on Sarcopenic Dysphagia. We initially included adults (aged ≥20 years) with limited oral intake, which was defined as a Food Intake Level Scale (FILS) [17] of ≤8.

### 2.2. Scale for Oral Intake Ability: Food Intake Level Scale (FILS)

The FILS is a 10-point observer-rating scale that measures the functional oral intake ability of the food type that the subjects ingest or the state of their nutritional intake. Level 10 represents no food ingestion restrictions, and level 1 represents no oral intake. From levels 1–3, subjects have no regular oral nutritional intake. From levels 4–6, subjects can ingest modified food that is easy to swallow with alternative/supplemental nutrition (e.g., tube feeding). From levels 7–10, subjects can ingest full nutrition orally. At level 7, subjects eat modified (easy to swallow) food, and at level 8, subjects eat regular food with some limitations of food items that are difficult to process (e.g., dry and brittle food or hard food) or thin drinks that are easy to aspirate. This index targets diseases such as stroke, neuromuscular diseases, and respiratory diseases with dysphagia, and the validation study mainly targeted elderly people in their 70s, which is similar to the subjects of the present study. The interrater reliability of FILS is reported to be 0.70–0.90 [17]. The accuracy of the FILS is assured by the standardization of the data entry process based on set guidelines.

### 2.3. Clinical/Care Settings and Registered Variables

Regarding the clinical/care setting in this study, patients from 17 facilities (9 acute and 8 recovery [convalescent] hospitals) were registered. The variables registered in the database were as follows: age, sex, ability of oral intake upon registration or admission (shown by the FILS level), causative disease upon admission, comorbidities, body function (walking speed, hand grip test, and five-times-sit-to-stand test if the data were available), calf circumference, whole-body sarcopenia diagnosed by the Asian Working Group for Sarcopenia (AWGS) 2019 criteria [18], the skeletal muscle mass index (SMI) measured by dual energy X-ray absorptiometry (DXA) or bioelectrical impedance analysis (BIA), maximum isometric tongue pressure, body mass index (BMI), presence of malnutrition in accordance with the Global Leadership Initiative on Malnutrition (GLIM) criteria [19], measures of nutritional intake, oral condition measured by the Revised Oral Assessment Guide (ROAG) [20] or Oral Health Assessment Tool (OHAT) [21], hoarseness, presence of dysarthria or aphagia, activities of daily living, and drugs upon admission. The follow-up was three months after registration or at discharge. At the time of follow-up, the variables collected were as follows: oral intake ability measured with the FILS, activities of daily living, and outcome (discharge to home, discharge to another hospital, continued hospitalization, or death).

### 2.4. Inclusion/Exclusion Criteria of the Study

Older adults (aged ≥60 years) who were registered in the JSDD comprised the final study population. The exclusion criteria were a lack of SMI data or an unknown measurement method of the SMI. In the present study, as we focused on the population whose SMI was estimated by BIA, the population whose SMI was estimated by DXA was excluded.

### 2.5. Sample Size Calculation

Assuming a mean difference between the two groups of 0.8, SD of 1.2, alpha error of 0.05, and power of 0.8 for this study, the required sample size would be 75 cases (Group A 30 cases and Group B 45 cases).

### 2.6. Statistical Analysis

Statistical analyses were performed using a free statistical software, EZR ver. 1.60 (Saitama Medical Center, Jichi Medical University, Saitama, Japan), which is a graphical user interface for R 4.2.1 (The R Foundation for Statistical Computing, Vienna, Austria). In the descriptive analysis of the patients’ background data, parametric data are expressed as the mean ± standard deviation (SD). Nonparametric data are expressed as the median and interquartile range. We divided the included population into two groups (low skeletal muscle mass group and high skeletal muscle mass group). The cut off value was decided with reference to previous studies. The cut off value of the SMI (measured with the BIA method) was decided as 6.5 kg/m^2^ for men and 4.98 kg/m^2^ for women. These cut off values were based on a study by Yamada et al. in 2014 [22] who evaluated SMI by the BIA method and studied healthy Japanese (men *n* = 16,379, women *n* = 21,660, mean age: 54.5 years). We set the cut off of this study at 2SDs below the mean SMI. The chi-square test, Mann–Whitney U test, and Student’s *t* test were used for comparisons between the groups. The correlation between the SMI upon admission and FILS at follow-up was analyzed using the Pearson correlation analysis. A strong correlation was defined as >0.70, a moderate correlation as 0.4 to 0.7, a weak correlation as 0.20 to 0.4, a negative correlation as <0.20. Bivariate and multivariate regression analyses for the FILS level at the follow-up were performed, and the crude and adjusted odds ratios were calculated. To adjust the odds ratio with the patient’s background disease that caused dysphagia (stroke and/or dementia), we calculated the propensity score and performed logistic regression analysis. We performed a post hoc test to evaluate the statistical power. The outcome variable (FILS) at the follow-up was set as a binary variable between levels 1–7 and levels 8–10. The significance level was set at *p* < 0.05.

The area under the ROC curve (AUC) of SMI from each sex upon admission were used to estimate the probability of the better FILS levels (levels 8–10) at the follow-up. The protocol of the present study was approved by the Ethics Committee of Tohoku University Graduate School of Medicine (approval number 2022-1-395 date of approval 27 July 2022).

## 3. Results

### 3.1. Included Population

**Figure 1:**
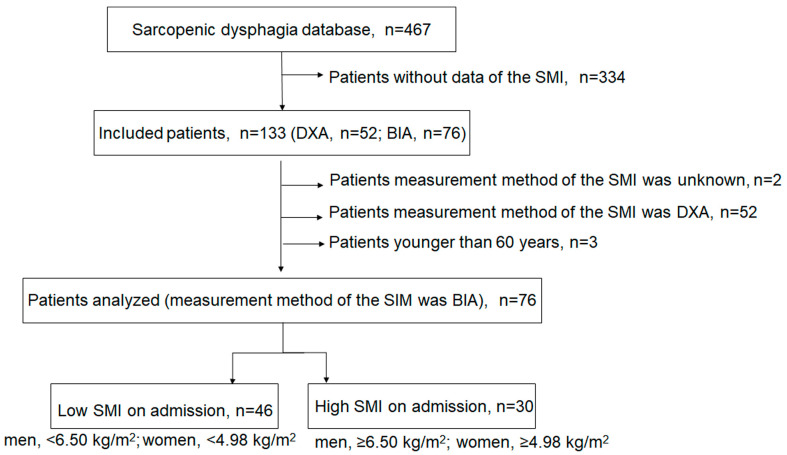
Study flow chart of patient inclusion.

Figure 1 shows a flow chart of the included population. The database comprised 467 patients with a FILS level of ≤8 at enrollment, and 76 patients were finally included from 133 patients with SMI data measured with BIA. We excluded patients whose measurement method of the SMI was DXA (*n* = 52), unknown (*n* = 2), and patients aged <60 years (*n* = 3). The patients were divided into two groups on the basis of the SMI. The low skeletal muscle mass group comprised 46 patients and the high skeletal muscle mass group comprised 30 patients.

### 3.2. The Patient Characteristics at Baseline

The patients’ characteristics at baseline are shown in Table 1. Among the patients, 61.8% (*n* = 47) were women, and the mean (SD) age upon admission or registration was 80.8 (9.0) years. The low skeletal muscle mass group comprised 46 patients (33 women and 13 men; mean [SD] age: 82.0 [7.7] years), and the high skeletal muscle mass group comprised 30 patients (14 women and 16 men; mean [SD] age: 79.1 [10.5] years). All patients included had their skeletal muscle mass measured by BIA. The median SMI in all patients was 5.0 kg/m^2^ (interquartile range: 4.5–6.5 kg/m^2^). The median SMI of each sex was 4.80 kg/m^2^ (interquartile range 4.10–5.10 kg/m^2^ [female gender]), and 6.50 kg/m^2^ (interquartile range 5.66–6.90 kg/m^2^ [male gender]), respectively. Upon admission, the median FILS level in all patients was 7 (interquartile range: 6–7). Regarding the causative disease upon admission in all patients, 22.4% (*n* = 17) had an ischemic stroke, 10.5% (*n* = 8) had an intracranial hemorrhage, and 31.6% (*n* = 24) had a hip fracture. Regarding the comorbidities, 17.1% (*n* = 13) had diabetes mellitus, 18.4% (*n* = 14) had a chronic heart failure, and 14.5% (*n* = 11) had a chronic kidney failure. Most (80.3%, *n* = 61) of the patients consumed their nutrition orally, and 18.4% (*n* = 14) of the patients used a nasogastric tube for nutrition intake. Using the GLIM criteria, more than half (*n* = 39, 51.3%) of the patients had severe malnutrition, and 43.4% (*n* = 33) had moderate malnutrition. Only 31 patients had data for maximum isometric tongue pressure, and the mean pressure (SD) was 15.8 (10.7) kPa. Regarding activities of daily living, 67 patients had the data of Barthel Index score, and the median score was 15.0 (interquartile range, 0–40). The median period from admission to follow-up of all patients was 79 (interquartile range 32–106.8) days.

### 3.3. Correlation between SMI upon Admission and FILS Level at the Follow-Up

**Figure 2:**
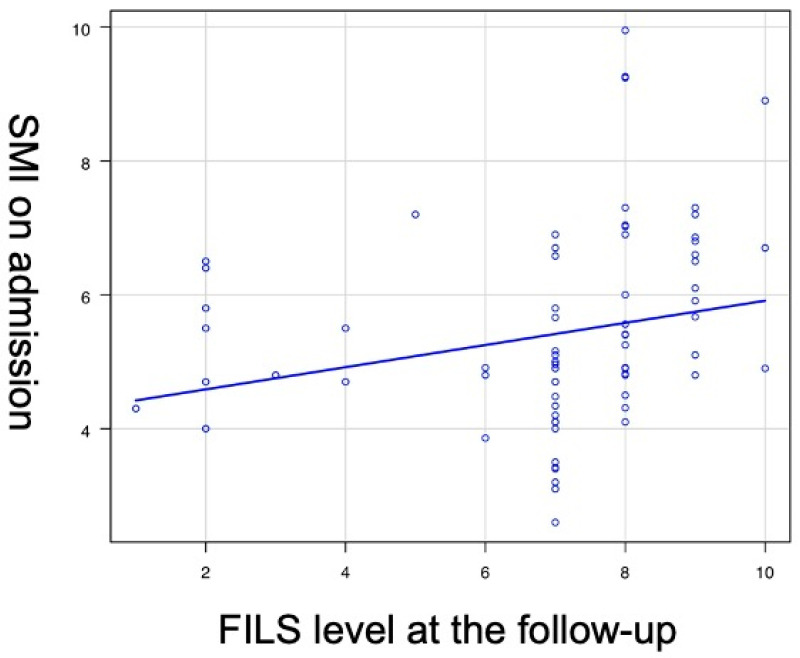
Correlation between SMI upon admission and FILS level at the follow-up. Each spot indicates the point of SMI on admission and FILS level at the follow up by each case. SMI: skeletal muscle mass index, FILS: Food Intake Level Scale, A week correlation was found between SMI upon admission and FILS level at the follow-up (r = 0.232, 95% CI 0.007–0.435, *p* = 0.04), Pearson correlation analysis. reflects the points for each case.

As shown in Figure 2, a weak correlation was found between SMI upon admission and FILS level at the follow-up (r = 0.232, 95% CI 0.007–0.435, *p* = 0.04).

### 3.4. Comparison between the High Skeletal Muscle Mass Group and the Low Skeletal Muscle Mass Group

We performed a comparison of the background characteristics between the high skeletal muscle mass group and the low skeletal muscle mass group (Table 1). There were no significant differences in age, FILS level upon admission, methods of nutrition upon admission, or maximum isometric tongue pressure between the two groups, whereas a significant difference was observed in sex *(p* = 0.033). Regarding the comorbidities, no statistical difference between the two groups was seen with respect to diabetes mellitus, a history of stroke and dementia. The low skeletal muscle mass group had a higher proportion of women than the high skeletal muscle mass group. BMI was significantly lower in the low skeletal muscle mass group than in the high skeletal muscle mass group (*p* < 0.001). The rate of the presence of malnutrition in accordance with the GLIM criteria was significantly higher in the low skeletal muscle mass group than in the high skeletal muscle mass group (*p* = 0.043). The activities of daily living score measured by the Barthel Index tended to be lower in the low skeletal muscle mass group than in the high skeletal muscle mass group, but this was not significant *(p* = 0.350). Regarding the period from admission to follow-up, no statistically significant difference was observed between the two groups (*p* = 0.133). The FILS level during follow-up was significantly different between the low skeletal muscle mass group and the high skeletal muscle mass group (median 7, interquartile range, 7–8, vs. median 8, interquartile range, 7–9, *p* = 0.008).

### 3.5. Bivariate and Multivariate Regression Analyses for the FILS Level at the Follow-Up

In the regression analysis for the FILS level (binary variable between levels 1–7 and levels 8–10) at the follow-up, the bivariate analysis showed that the female sex was associated with a 1.98 times (crude odds ratio) higher risk of a FILS level of 1–7 than the male sex, but this difference was not significant (95% confidence interval [CI]: 0.78–5.07) (Table 2). We also found that patients with a history of stroke and/or dementia had a 69.7 times (crude odds ratio) higher risk of a FILS level of 1–7 at the follow-up than those without a history of stroke and/or dementia (95% CI: 0.75–6500.00), but this was not statistically significant. The stroke history ‘s pure effect to lower the FILS level at the time of follow-up was 0.88 times (crude odds ratio) (95% CI: 0.49–1.57, *p* = 0.66). The low SMI group had a 3.57 times (crude odds ratio) higher risk of not achieving adequate oral intake than that of the high SMI group (95% CI: 1.36–9.37, *p* < 0.01).

After adjusting for age, sex, and patient’s history of stroke and/or dementia in the multivariate regression analysis, the SMI upon admission (odds ratio: 2.99, 95% CI: 1.09–8.16) was significantly associated with the FILS level at the follow-up (*p* = 0.033). We did not include maximum isometric tongue pressure and the Barthel Index upon admission in the logistic regression model because of missing values. A low skeletal muscle mass upon admission indicated a 2.99 times greater chance that a patient would have an oral intake ability of a FILS level of <7 at the subsequent 3-month follow-up.

Results of the post hoc test showed the statistical power of 0.756 (ratios on the low skeletal muscle mass and high skeletal muscle mass groups: 0.674, and 0.367, respectively; α error 0.05, two-sided test).

### 3.6. The ROC Curve Analysis

**Figure 3:**
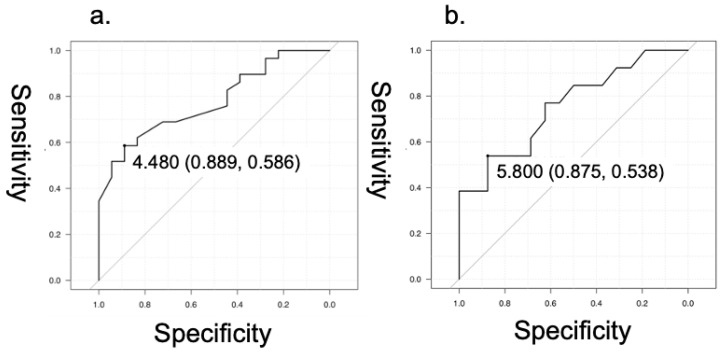
ROC curves of SMI upon admission for higher FILS levels (levels 8–10). (**a**) ROC curve for women, AUC 0.778 (95% CI 0.647–0.908). The best threshold to discriminate FILS levels at the follow-up, 4.480 kg/m^2^. (**b**) ROC curve for men, AUC 0.775 (95% CI 0.574–0.936). The best threshold to discriminate FILS levels at the follow-up, 5.800 kg/m^2^.

The AUC was used as a measure to predict accuracy for oral intake ability at the follow-up (Figure 3a,b). The AUC of female SMI upon admission was 0.778 (95% CI 0.647–0.908). The most predictive threshold was 4.480 kg/m^2^ for females (sensitivity 0.889, specificity 0.586). The AUC of male SMI upon admission was 0.775 (95% CI 0.574–0.936). Both AUC for females and males indicated intermediate accuracy (>0.70). The most predictive threshold was 5.800 kg/m^2^ for males (sensitivity 0.875, specificity 0.538). Thresholds obtained for both female and male patients have relatively high sensitivity and low specificity.

## 4. Discussion

We performed a retrospective cohort study to determine the relationship between the SMI measured with BIA and oral intake ability 3 months after admission at a participating hospital. This study showed two novel findings. The first finding was that a low skeletal muscle mass affected subsequent swallowing ability in older adults hospitalized in acute and convalescent wards. The second finding was that the degree of association between a low skeletal muscle mass and the oral intake ability in the participating patients was 2.99 times higher than that in those without a low muscle mass.

In our study, a low skeletal muscle mass affected subsequent swallowing ability in older adults hospitalized in acute and convalescent wards. From the coefficient analysis, a weak but statistically significant correlation was found between SMI upon admission and FILS level at the follow-up (r = 0.232, 95% CI 0.007–0.435, *p* = 0.04). Maeda and Akagi showed that the SMI was an independent factor for the prevalence of dysphagia in hospitalized older people in a retrospective, cross-sectional study [23]. The present study also showed a causative relationship between skeletal muscle mass and oral intake ability. Maeda et al. showed a relationship between SMI and dysphagia 60 days after admission in hospitalized older adults who stopped oral intake 2 days after admission [24]. However, their study was performed in only one facility. The study also had a potential limitation in that it showed causality between the SMI upon admission and dysphagia because inhibition of oral intake itself can affect swallowing ability. In the present study, we showed a relationship between skeletal muscle mass and swallowing ability using data from multiple centers without a period of oral intake prohibition.

The extent of the association between a low skeletal muscle mass and the oral intake ability in older adults was 2.99 times higher than that in older adults without a low skeletal muscle mass. The post hoc test of this statistical analysis showed a fair statistical power (0.756) regardless of the relatively small sample size. Maeda et al. [24] reported that the odds ratio of a low SMI being associated with dysphagia (≤level 5 on the Functional Oral Intake Scale) [25] was 24.0 (95% CI: 3.6 to 159.0). This value was higher than that in our study. The reasons for this difference between studies may be that Maeda et al.’s finding was affected by 48 h of prohibited oral intake in all patients. Maeda and Akagi [23] also reported that the adjusted odds ratio of a high SMI for the presence of dysphagia (measured with the Functional Oral Intake Scale) was 0.48 (95% CI: 0.328 to 0.712). These authors performed a subgroup analysis for a low SMI (<7.0 kg/m^2^ for men and <5.7 kg/m^2^ for women), and the effect size of the SMI in men was 0.4 and that in women was 0.3. The odds ratio of 2.99 in the present study reflected the risk of low skeletal muscle mass affecting the subsequent oral intake ability without the inhibition of oral intake during the observation period.

The cutoff for the SMI in the present study for men and women was set at 2 SDs below the mean SMI for middle-aged, healthy, Japanese people (mean [SD] age: 54.5 [9.9] years [22]). To determine the cut off value, we referred to the system of the Asia Working Group for Sarcopenia 2019 criteria [18] and the Asia Working Group for Sarcopenia 2014 [26], which recommends using 2 SDs below the mean muscle mass of the young reference group. We consider 2SDs below the mean appropriate because the difference between the mean age in the present study (80.8 years) and the reference groups was approximately 25 to 30 years. The ROC curve analysis indicated the best cutoff value of SMI upon admission to differentiate FILS levels at the follow-up as 4.480 kg/m^2^ for females, and 5.800 kg/m^2^ for males. These are relatively close to the cutoff value authors set before analysis.

In older patients in acute and convalescent wards who eat regular food with some limitations, if skeletal muscle decreases to <2 SDs of the value at baseline, the risk of oral intake ability requiring dietary modification (FILS ≤ 7) can increase 2.99-fold after 3 months. We speculate that skeletal muscle mass affected the subsequent oral intake ability through mastication, endurance of the masticatory muscles, and endurance of the trunk muscles to maintain posture during a meal. Masseter muscle thickness is associated with oral phase dysphagia in institutionalized older individuals [27]. To determine the effect of mastication, we set the cut off value for the FILS between levels 7 and level 8, because patients must masticate their meals at these levels.

This study suggested that a low skeletal muscle mass played a major role in the subsequent oral intake ability in older patients with limited oral intake upon admission. Previous studies have shown that older adults who eat a texture-modified diet tend to have poor appetite [28]. Poor appetite may be a cause or a consequence of reduced muscle mass and oral intake. When nutritional intake is insufficient, muscle is broken down from the body’s stored protein (catabolism) to use as an energy resource. People who have a low muscle mass upon admission to the hospital may have less reserve (endogenic energy resource) than that in people who have sufficient muscle mass. Additionally, people with a low muscle mass may not be able to compensate for this decrease because of insufficient nutritional intake with a texture-modified diet. Nakahara and colleagues reviewed aggressive nutritional therapy and proposed the use of this therapy in sarcopenic patients [29]. Aggressive nutritional therapy was defined as nutritional management in which daily energy expenditure was considered, and the amount of energy accumulation was set as the energy requirement [29]. To prevent a further decline in oral intake ability, aggressive nutritional therapy can be indicated when setting a goal of energy intake for the aged population with already limited oral intake.

There are several limitations to this study. First, the food type that was provided to the patients might not have reflected their true deglutition ability. Unfortunately, the database did not include the data that reflect the actual swallowing function measured using video endoscopy, modified barium swallow examination, or water swallow screening examination. Thus, in the present study, we assessed the oral intake ability by the food type, which might have been determined by the primary doctors, nurses, or caregivers. Caregivers might have been tempted to change the type of the food so that patients could process it easier as there is a low requirement for endurance of the mastication muscles. Masticatory muscle endurance may be associated with a reduced skeletal muscle mass, regardless of the patients’ deglutition ability. Patients with a FILS level ≤ 7 might not have had adequate dentition or appropriate dentures, which is required for mastication of nuts or rice cakes, and these patients may have been able to ingest only limited food types. Second, this study analyzed patients with adjustment of diseases that can be a clear cause of dysphagia. However, not all diseases or social backgrounds that can influence the oral intake ability were controlled. Third, this study did not provide detailed information on BIA’s measurement condition as that information was not collected at the time of registration on this database. We asked for this information retrospectively from the registered facilities by online questionnaire. Six facilities answered the questionnaire. All the facilities that answered the questionnaire measured the BIA data with direct segmental multifrequency BIA (InBody^TM^, Cerritos, CA, USA). As for the timing and measurement conditions, one facility measured before mealtime, one facility measured >2 h after mealtime, and one facility had a rule to measure 15 min after bed rest time to stabilize the body fluid. Additionally, two facilities had no rule related to timing and measurement condition. Fourth, the time to follow-up varied by case. The median follow-up period was 79 days, ranging from a minimum of 7 days to a maximum of 229 days. This could affect the results of the present study. The last limitation was the absence of the data on appetite. Appetite is an important aspect to consider in relation to the prognosis of oral intake ability. Unfortunately, the data on appetite were not included in the current database, thus, consequently, we could not perform a relevant analysis.

## 5. Conclusions

This study shows that a low skeletal muscle mass affects the subsequent oral intake ability among the aged population with limited oral intake upon admission. Our findings indicate that a low skeletal muscle mass can be a disadvantage to acquiring subsequent fully functional oral intake ability in older adults with limited oral intake upon admission. This population should be encouraged to take all possible measures to promote nutritional intake to prevent a further decline in their oral intake ability on and after hospital discharge. More evidence is required regarding the relationship between skeletal muscle mass and dysphagia.

## Figures and Tables

**Table 1:** Patients’ characteristics at baseline and results of the bivariate analysis of the skeletal muscle mass index (SMI) data.

Variable	Total *n* = 76	Low SMI upon Admission *n* = 46	High SMI upon Admission *n* = 30	*p*-Value
Female, *n* (%)	47 (61.8)	33 (71.7)	14 (46.7)	0.033 ^+^
Male, *n* (%)	29 (38.2)	13 (28.3)	16 (53.3)	
Age upon admission, mean (SD)	80.8 (9.0)	82.0 (7.7)	79.1 (10.5)	0.172 ^@^
FILS upon admission, median (interquartile range)	7 (6–7)	7 (6–7)	7 (5–8)	0.416 *
Causative disease upon admission				0.219 ^+^
Ischemic stroke *n* (%)	17 (22.4)	7 (15.2)	10 (33.3)	
Intracranial hemorrhage *n* (%)	8 (10.5)	3 (6.5)	5 (16.7)	
Subarachinoid hemorrhage *n* (%)	1 (1.3)	0 (0.0)	1 (3.3)	
Hip fracture (ICD 10 code S7200 or 7210), *n* (%)	24 (31.6)	16 (34.8)	8 (26.7)	
Heart failure, *n* (%)	5 (6.6)	3 (6.5)	2 (6.7)	
Vertebral fracture, *n* (%)	5 (6.6)	4 (8.7)	1 (3.3)	
Other fracture, *n* (%)	1 (1.3)	1 (2.2)	0 (0.0)	
Disuse, *n* (%)	7 (9.2)	5 (10.9)	2 (6.7)	
Other disease than above, *n* (%)	8 (10.5)	7 (15.2)	1 (3.3)	
Comorbidities				
Cancer, *n* (%)	7 (9.2)	4 (8.7)	3 (10.0)	1.00 ^+^
Diabetes Mellitus, *n* (%)	13 (17.1)	6 (13.0)	7 (23.3)	0.351 ^+^
Chronic Heart Failure, *n* (%)	14 (18.4)	8 (17.4)	6 (20.0)	0.772 ^+^
Chronic Kidney Failure, *n* (%)	11 (14.5)	7 (15.2)	4 (13.3)	1.00 ^+^
Chronic Obstructive Pulmonary Disease, *n* (%)	1 (1.3)	0 (0.0)	1 (3.3)	0.395 ^+^
History of Stroke, *n* (%)	14 (18.4)	6 (13.0)	8 (26.7)	0.225 ^+^
Dementia, *n* (%)	26 (34.2)	17 (37.0)	9 (30.0)	0.624 ^+^
Method of nutrition Intake				
Oral intake, *n* (%)	61 (80.3)	38 (82.6)	23 (76.7)	0.565 ^+^
Nasogastric tube, *n* (%)	14 (18.4)	7 (15.2)	7 (23.3)	0.384 ^+^
PEG, *n* (%)	0 (0.0)	0 (0.0)	0 (0.0)	NA
Peripheral parenteral nutrition, *n* (%)	11 (14.5)	6 (13.0)	5 (16.7)	0.744 ^+^
BMI (kg/m^2^), mean (SD)	20.3 (3.7)	18.9 (3.1)	22.4 (3.7)	<0.001 ^@^
Malnutrition in accordance with the GLIM criteria				
No malnutrition, *n* (%)	4 (5.3)	0 (0.0)	4 (13.3)	0.043 ^+^
Moderate, *n* (%)	33 (43.4)	22 (47.8)	11 (36.7)	
Severe, *n* (%)	39 (51.3)	24 (52.2)	15 (50.0)	
Maximum isometric tongue pressure (kPa), mean (SD), *n* (%)	15.8 (10.7) *n* = 31	14.5 (10.4) *n* = 22	19.0 (11.6) *n* = 9	0.299 ^@^
Poor oral condition with OHAT ^￥^, (*n* = 4) *n* (%)	1 (1.3)	1 (2.2)	0 (0.0)	1.000 ^+^
Poor oral condition with ROAG ^￥^, (*n* = 72) *n* (%)	69 (98.6)	43 (93.5)	26 (86.7)	0.424 ^+^
Barthel Index (*n* = 67) median (interquartile range)	15.0 (0–40)	10.0 (0–27.5)	15.0 (2.5–40)	0.350 *
Period from admission to follow-up (days) median (interquartile range)	79 (32–106.8)	73.5 (30.5–88.75)	85.5 (40.35–137.75)	0.133 *

SMI: skeletal muscle mass index, FILS: Food Intake Level Scale, ICD 10: International Classification of Diseases 10th edition, PEG: Percutaneous Endoscopic Gastrostomy, BMI: body mass index, GLIM: Global Leadership Initiative on Malnutrition, SD: standard deviation. ^￥^ Revised Oral Assessment Tool (ROAG) ≥ 9, Oral Health Assessment Tool (OHAT) ≥ 7: poor oral condition. ^+^ Chi-square test; * Mann–Whitney U test; ^@^ Student’s *t*-test.

**Table 2:** Bivariate and multivariate regression analyses of the Food Intake Level Scale (FILS) data at the follow-up.

*n* = 76	Bivariate		Multivariate	
Variable	Crude Odds Ratio	95% CI	Adjusted Odds Ratio	95% CI
Female	1.98	(0.78–5.07)	1.37	(0.41–4.61)
Age upon admission	1.02	(0.97–1.07)	0.99	(0.92–1.06)
Propensity Score: Stroke and Dementia	69.7	(0.75–6500.00)	21.3	(0.09–5080.00)
BMI	0.89	(0.78–1.01)		
Maximum isometric tongue pressure (*n* = 31)	0.95	(0.89–1.02)		
Barthel Index upon admission (*n* = 67)	0.97	(0.95–0.99) *		
SMI upon admission	3.57	(1.36–9.37) **	2.99	(1.09–8.16) *

CI: confidence interval, SMI: skeletal muscle mass index, FILS: Food Intake Level Scale, BMI: body mass index, * *p* < 0.05; ** *p* < 0.01, power = 0.756.

## Data Availability

Data sharing not applicable—no new data generated.
